# Gut microbiota variability in dung beetles: prokaryotes vary according to the phylogeny of the host species while fungi vary according to the diet

**DOI:** 10.3389/finsc.2025.1639013

**Published:** 2025-08-20

**Authors:** Gianluca Natta, Samuele Voyron, Erica Lumini, Alex Laini, Angela Roggero, Alessandro Fiorito, Claudia Palestrini, Antonio Rolando

**Affiliations:** ^1^ Department of Life Sciences and Systems Biology (DBIOS), University of Turin, Turin, Italy; ^2^ Institute for Sustainable Plant Protection (IPSP) – National Research Council (CNR), Turin, Italy

**Keywords:** bacteria, coprophagy, ecological functions, functional similarity, fungi

## Abstract

Dung beetles (Coleoptera, Scarabaeoidea) support several ecological processes and services making them important ecosystem engineers. The dung beetle gut microbiota is involved in many of these ecological services. In the present study, we analyzed the microbiota of 90 individuals of three *Onthophagus* species feeding on different dung types. Our aim was to understand whether the species identity affected the microbiota more than the dung ingested and whether this conditioning applied equally to prokaryotes and fungi. We also compared the taxonomic and functional variability of the microorganisms to check for similarities between individuals. Using molecular analyses, we characterized the alpha and beta diversities, core and indicator taxa and taxonomic and functional composition of the gut microbiota. Alpha diversity analyses revealed diet, species and sex to influence diversity parameters but no clear differences in the diversity patterns for prokaryotes vs fungi. Conversely, all other analyses consistently showed differences in the composition patterns for prokaryotes vs fungi, with prokaryotes mostly varying according to host species identity and fungi varying according to dung type. This suggests that most prokaryotes in the dung beetle microbiota are definitive symbionts, whereas many fungi are transient symbionts. We found evidence of great similarity in the functional composition of the microbiota despite strong taxonomic dissimilarities. The results emphasize the need to consider both the prokaryotic and fungal components of the microbiota. They also suggest microbial composition analyses to be preferable to alpha diversity analyses for identifying patterns of variation that depend on phylogeny and diet.

## Introduction

Dung beetles (Coleoptera, Scarabaeoidea) can be considered important ecosystem engineers, being involved in numerous ecological services and processes such as dung removal, soil fertility maintenance, nutrient cycles such as nitrogen (N) and carbon (C) cycling, and the control of greenhouse gas emissions (1–5). Previous studies have shown both the growth and development of dung beetles to be influenced by their gut microbiota (6, 7). For instance, these microbes facilitate digestion by providing essential enzymes which protect beetles against certain pathogens (8–11). At the same time, many of the ecological services associated with dung beetles are actually provided by symbiotic microorganisms, the action of which may depend on the behavior and ecology of their hosts. For example, the tunnel-digging behavior of certain dung beetles favors the aeration and drying of the dung, leading to a reduction in methane emissions as methanogenic prokaryotes are negatively affected by dung oxygenation and desiccation (12, 13).

Studies have demonstrated that part of the dung beetle microbiota has a vertical parental derivation (14, 15). Females of the genus *Onthophagus* emit and then lay their eggs upon a fecal secretion called a pedestal (14), which the larvae then feed upon after hatching, and this transmission of maternal microbiota is essential for the survival and proper development of the larvae (6, 16). Females of *Catharsius molossus* do not deposit a pedestal, instead the larvae feed on the inner soil layer of the brood ball after hatching, and this layer (called the ‘parental gift’) may similarly serve as a medium for the vertical transmission of microbes (11). In the endemic Australian genus *Cephalodesmius*, males and females work together to gather dung, carrion, fungi, leaves, fruits and flowers to form a brood mass of composting material. Adult feces are added to the mass, in essence, inoculating it with hindgut microbiota (15).

The gut microbiota is essential for the survival of dung beetles, as shown by switch and transplant experiments. Switching the pedestal deposited by *Onthophagus gazella* which that from *O. sagittarius* resulted in delayed development and higher mortality (6); whereas transplanting the microbiota of syntopic *O. vacca* into *O. medius* [and vice versa] caused various developmental and survival problems, despite their being sister species (17). Similar results were obtained in an experiment conducted in two other sister species, namely *O. taurus* and *O. illyricus* (18).

Whilst several studies have suggested different species to be characterized by distinct microbiota compositions, intraspecific microbial variability is also very high. Considerable taxonomic differences in the microbiota of wild individuals were highlighted in a population of *Trypocopris pyrenaeus*, which was probably dependent on the type of dung consumed by the individuals (19). The two sexes may also present significant differences, as found in *Euoniticellus intermedius* (20).

The structure and composition of the dung beetle gut microbiota may depend on phylogeny (i.e. the taxonomic distances between species), diet and gut morphology (15, 20–22). Phylogenetic differences, evaluated by considering host species that are (to varying extents) taxonomically distant, seem to have a relevant effect on both the composition and structure of microbial communities. Indeed, two studies addressing Scarabaeinae beetles showed phylogeny (evaluated by considering dung beetles of five different tribes) to be a bigger driver of gut microbiota taxonomic composition than habitat and dietary factors (15, 22). Analogously, relevant differences in alpha diversity metrics were even found between closely related species living in the same habitat and eating the same type of diet; for example, considering coprophagous species only, the microbiota of *Aphodius depressus* was eight times more diverse than that of *A.* sp*hacelatus*, and that of *Onthophagus taurus* was on average four times more diverse than that of *O. ovatus* (21).

It is important to note that all of the results mentioned above solely addressed the prokaryotic gut microbiota; however, the gut microbiota of dung beetles also includes fungi in addition to bacteria and archaea (10, 15, 19, 22, 23). Nevertheless, only a few studies to date have investigated both prokaryotes and fungi contemporaneously (11, 19, 23). Recent previous research highlighted the importance of considering both the prokaryotic and fungal components of the microbiota as values of host individual ordination (nMDS) changed significantly depending on whether fungi or bacteria were considered (19). As far as we know, the potential causes of fungal variability in the gut microbiota have yet to be explored in depth, as have the effects of dung beetle diet on the microbiota. Indeed, research investigating the effects of dung beetle diet on gut microbiota composition has, so far, primarily taken diet in its broadest sense into consideration, i.e. whether a species is coprophagous, necrophagous or mycophagous, etc. (15, 23, 24), or the effects of feeding on dry vs wet dung only (10, 20, 23). However, diets can vary greatly within each of these broad categories; for instance, coprophagous beetles can exploit the dung released by many different species of vertebrates, especially mammals, according to their preferences (25–28) and local availability. Therefore, to understand how dietary variety can influence the composition of the microbiota, it is essential to focus on the potential consequences resulting from the use of excrement deposited by one mammal species or another. Although the movements of dung beetles in their environment have not been studied in detail, certain investigations indicate that most individuals move between dung pats of the same pasture and that movements between pastures decrease exponentially with distance (29). Furthermore, when beetles reproduce, it is reasonable to assume that they feed on that type of dung for quite a long time, especially if reproduction involves the construction and defense of the nest and, possibly, prolonged parental care (30). These considerations suggest that, to study the potential effect of different types of dung on the gut microbiota, it is sufficient to collect dung beetles feeding on droppings located in spatially distant pastures being grazed by distinct species of mammals (for example, different species of livestock), i.e. pastures characterized by a supply of different dung types. At the same time, when faced with a very varied diet, we must also consider the great ability of the gut microbiota to respond to different food intakes without resulting in substantial changes to the functions performed. This concept introduces the idea that, up to a certain point, microbes, especially prokaryotes are interchangeable in terms of their function (31, 32). It has been suggested that a ‘functional’ rather than ‘taxonomic’ core microbiota may be more informative in determining gut microbiota composition (33, 34). Such functional similarity has also been observed in previous studies on the dung beetle species *Pachysoma* spp. and *T. pyrenaeus* (19, 23). Thus, studying and comparing the microbiota of hosts with different diets can enhance our understanding of the functional roles of various gut microbiota by enabling us to assess whether these functions remain stable despite changes in the ingested food.

In the present study, we examined both the prokaryotes and the fungi present in the gut of wild adult dung beetles that had been collected in different types of dung from distinct pastures. We focused on three closely related species belonging to the same genus (*Onthophagus* Latreille, 1802) and subgenus (*Palaeonthophagus* Zunino, 1979). Our aim was to understand whether, in the case of phylogenetically very close species, species identity affected the microbiota more so than the dung ingested and whether this conditioning applied to prokaryotes and fungi equally. Another objective was to compare taxonomic and functional variability to check for functional similarity between individuals.

## Materials and methods

### Species collection and environmental sampling

Ninety (N=90) adult individuals of three species of the genus *Onthophagus* (*Palaeonthophagus*) were collected in the Western Italian Alps [Susa valley, Condove (TO), Piedmont (45.136° N, 7.296° E)] in May 2023. The three species, namely *O. fracticornis* (OFT), *O. medius* (OMD) and *O. verticicornis* (OVT), were identified considering their morphological traits (35) and through molecular characterization, using the mitochondrial cytochrome C oxidase subunit I (COI) gene sequence as a DNA barcode (**Supplementary File 1**, **Supplementary Table S1**).

We collected 30 individuals (15 males and 15 females) of each species: 10 from cow dung, 10 from sheep dung and 10 from donkey dung, with each dung type located in a distinct pasture. Two soil and two dung samples were collected from each of the three pastures, where cattle, sheep, and donkeys were being grazed, respectively: two dung pats from each pasture were chosen at random, and the soil samples were obtained from approximately 5 cm below the surface of the ground located next to these dung pats. The dung and soil samples were stored in 1.5 ml Eppendorf tubes. The two samples of the same dung or soil were subsequently homogenized in the laboratory prior to analysis. Absolute ethanol was added to the tubes containing dung samples to prevent dung fermentation. All samples were then stored at -20°C.

### Gut removal

Dung beetles were housed in plastic terraria without food for at least 24 hours to make them excrete as much ingested dung as possible and thus clean out their guts. Individuals were euthanized by submersion in absolute ethanol and immediately dissected to extract the entire gut. The dissection tools were sterilized using a 30% sodium hypochlorite solution and then washed in distilled water. Once removed, the gut was preserved in absolute ethanol and stored at 4°C.

### DNA extraction, amplification and Illumina NovaSeq sequencing

We extracted DNA from gut and dung samples using the CTAB modified method described in Natta et al. (19), while DNA from soil samples was extracted using the DNeasy^®^ PowerSoil^®^ Pro Kit (QIAGEN). A DNA metabarcoding approach was used to investigate microbiota: for the prokaryotic component, the 16S ribosomal RNA (rRNA) gene was amplified using the primer set 515fB (5′–GTGYCAGCMGCCGCGGTAA–3′) (36) and 806rB (5′–GGACTACNVGGGTWTCTAAT–3′) (37); for the fungal component, the nuclear ribosomal ITS2 region was amplified using the primer pair fITS7 (5′– GAACGCAGCRAAIIGYGA–3′) and ITS4 (5′–TCCTCCGCTTATTGATATGC–3′) (38). The following Illumina overhang adapter sequences were added to the primer pairs: forward overhang: 5′-TCGTCGGCAGCGTCAGATGTGTATAAGAGACAG-[locus specific target primer]; reverse overhang: 5′-GTCTCGTGGGCTCGGAGATGTGTATAAGAGACAG-[locus specific target primer].

PCR reactions were run in a final volume of 25 μl using 1 U of XtraTaq Pol White DNA polymerase (GeneSpin Srl, Milano, Italy), 5x XtraWhite Buffer with MgCl_2_, 0.2 μM of each dNTP, 0.5 μM of each primer and 20 ng of genomic DNA. For the prokaryotic community, the PCR cycling program consisted of an initial step at 94°C for 3 min, 35 cycles at 94°C for 45 s, 55°C for 60 s and 72°C for 90 s, followed by a final extension step of 72°C for 10 min. For the fungal community, the PCR cycling program consisted of an initial step at 94°C for 5 min, 35 cycles at 94°C for 30 s, 50°C for 30 s and 72°C for 30 s, and a final extension step of 72°C for 7 min.

Extracted DNA was amplified in triplicate and pooled before purification using Wizard^®^ SV Gel and the PCR Clean-Up System (Promega). PCR purified products were quantified using the Qubit dsDNA BR Assay kit and Qubit Fluorometer 2.0 following the manufacturer’s protocol and sent to IGA technologies (Udine, Italy) for Illumina NovaSeq sequencing (2 × 250 bp).

### Bioinformatics and statistical analysis

The following bioinformatics and statistical analyses were conducted for prokaryotes (i.e. archaea and bacteria) and fungi separately. Sequencing adapters and primers were removed, and then the sequences were analyzed using the microbiome bioinformatics platform QIIME2 (Quantitative Insights Into Microbial Ecology 2, v. 2021.2 (39). Denoising and quality control, including chimaera removal, were performed using the DADA2 plugin (40) through the qiime dada2 denoise-paired command, with chimaera detection carried out using the “consensus” method. The taxonomic assignment of the prokaryotic community was achieved using the Silva 138 99% OTUs full-length sequences database (41, 42), whereas for fungi we used the UNITE Community (2019): UNITE QIIME release for fungi v.04.04.2024 (43). Phylogenetic trees were generated by the QIIME2 plugin “qiime phylogeny align-to-tree-mafft-fasttree”. The outputs of the QIIME2 pipeline “taxonomy.qza”, “otu_table.qza” and “rooted-tree.qza”, together with their metadata files, were then imported into Rstudio (44) to create phyloseq objects using the R package qiime2R v.0.99.6 (45). Shared amplicon sequence variants (ASVs) between the beetles and the environmental samples (i.e., dung and soil samples) were then sought by analyzing the results of the flower plots generated from all 96 samples, using the function *plot_venn* in the R package microeco (46). The shared ASVs were removed from the phyloseq object.

Following the removal of shared ASVs, we used the phyloseq objects for the following diversity analyses. To allow for the comparisons of samples with non-uniform coverage, we normalized the ASV tables using the *rarefy_even_depth* function of the R package phyloseq v.1.36.0 (47). We used the *rarecurve* function of the R package vegan v2.6-2 (48) to obtain rarefaction curves of the rarefied ASV table. From the ASV table, we calculated absolute counts and relative abundances (i.e. the ratio between the number of reads belonging to the ASV in a specific sample and the total number of reads in the sample) for each ASV.

We evaluated alpha diversity using “Observed ASVs”, “Shannon” and “Faith’s Phylogenetic Distance (Faith PD)” indices using the *estimate_richness* function of the R package phyloseq. We then tested the effect of species, dung type, sex and their interaction on alpha diversity by analysis of variance (ANOVA). We checked the normal distribution of the model dataset by performing the Shapiro-Wilk test on the residuals. This analysis was not performed on the Observed ASVs because of the highly positive correlation with Shannon and Faith PD.

For both prokaryotes and fungi, we visualized dissimilarity between individuals by means of non-metric multidimensional scaling (nMDS) based on a Bray-Curtis distance matrix of ASV composition using R package vegan. Stress was used as a measure of goodness of fit. We plotted the results of the nMDS using the tidyverse collection of R packages (49), first in a general plot for all 90 individuals and then by dividing the plot into three distinct sub-plots to reveal any differences better, considering the 30 individuals belonging to each of the three species or collected in the three dung types. We performed an *envfit* analysis on the nMDS ordination to determine which of the factors had the greatest influence in differentiating individuals based on the R^2^ values and p-values.Differentiating microbial variability due to dung preference from that due to species is very difficult because these two factors were inseparably associated (we collected the individuals of a species in a certain type of dung). Therefore, the best way to study them was to investigate the interindividual variability in all possible associations between each species and each type of dung. To do so, we considered the Cartesian product of two sets, A and B, which consists of all the ordered pairs that can be constructed with the first element coming from the first set, A, and the second element coming from the second set, B. In our case, one set comprised the three *Onthophagus* species, and the other set comprised the three dung types. The 9 pairs (3 species x 3 dung types) were therefore: OFT-cow, OFT-sheep, OFT-donkey, OMD-cow, OMD-sheep, OMD-donkey, OVT-cow, OVT-sheep and OVT-donkey. We considered these 9 pairs in the analyses of shared, exclusive and indicator ASVs, and in taxonomic and functional analyses of the gut microbiota of dung beetles.

To visualize the shared (i.e. core) and exclusive ASVs of the nine pairs, we used flower plots generated using the R package microeco (46). In addition, we investigated the indicator ASVs of each of the nine pairs using the *multipatt* function in the R package indicspecies (50). To visualize the taxonomic diversity of the different samples, we used percent stacked bar charts generated using the tidyverse collection of R packages (49).

The trophic behaviors of the identified communities of prokaryotes and fungi were assessed using FAPROTAX (Functional Annotation of Prokaryotic Taxa) v. 1.2.6 (51) and FUNGuild (52), respectively, both of which were implemented in the R package microeco (46). As done for the taxonomic diversity, we used percent stacked bar charts to visualize the differences in trophic behavior between the samples. The effect of species and dung type on taxonomic and functional composition was tested using ANOVA and the Tukey honestly significant difference (HSD) test.

## Results

### Phylogenetic relationships of the three dung beetle species

COI sequences confirmed that the three species (OFT, OMD and OVT) were correctly identified using external morphological traits. All three species considered in this work fell into the same cluster, namely the subgenus *Palaeonthophagus* (**Supplementary File 1**, **Supplementary Figures S1**, **S2**).

### Alpha diversity

The bioinformatics analysis gave rise to 4659 prokaryotic ASVs and 4549 fungal ASVs. The “Observed ASVs” rarefaction curves showed that all 90 beetle samples reached the asymptote for both prokaryotes and fungi. Thus, the depth of sequencing was adequate to represent the diversity of the gut microbiota (**Supplementary Figures S3A, B**). The rarefied number of reads per sample was 1193 for prokaryotes and 1901 for fungi.

Regarding the prokaryotes, ASVs ranged from a minimum of 24 in a male OMD individual collected from donkey dung to a maximum of 395 in a male OMD collected from cow dung (**Supplementary Table S2**). We found significant differences in the Shannon index based on sex (F = 4.37, p < 0.05) and the interaction term sex-dung type (F = 4.12, p < 0.05). Significant differences were also highlighted in the Faith PD index based on the interaction term species-sex (F = 5.02, p < 0.01) and dung type (F = 3.40, p < 0.05).

Regarding the fungi, the number of ASVs detected ranged from 20 in a male OFT individual collected from donkey dung to a maximum of 399 in a male OMD, once again collected from donkey dung (**Supplementary Table S3**). The Shannon index varied significantly according to dung type (F = 11.75, p < 0.001), the interaction term species-sex-dung type (F = 5.12, p < 0.01), and on the interaction term species-sex (F = 4.26, p < 0.05). We found significant differences in the Faith PD index based on dung type (F = 8.64, p < 0.001) and the interaction term species-sex-dung type (F = 4.36, p < 0.01).

### Beta diversity: ordination by nMDS

**Figure 1:**
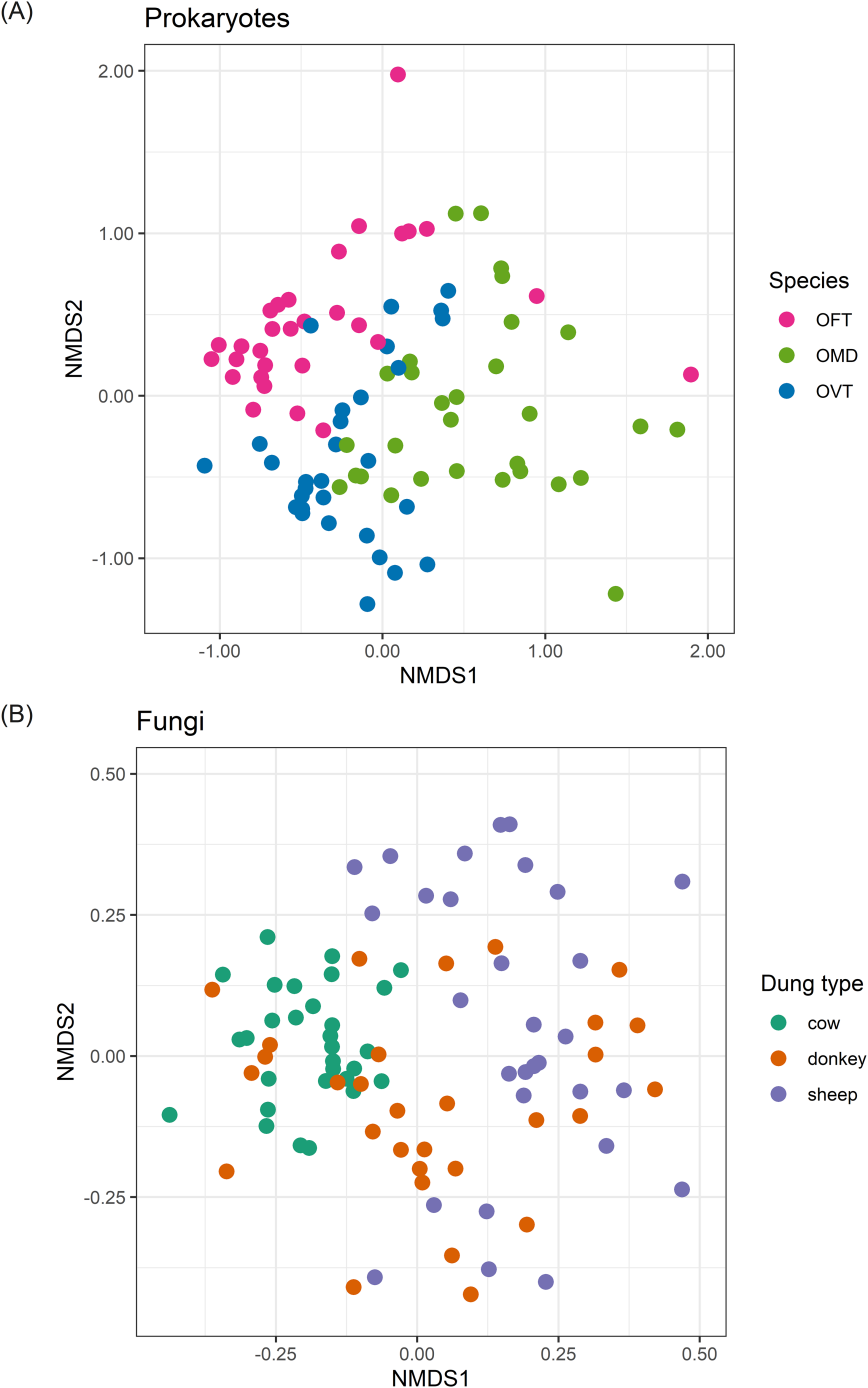
nMDS ordination plots for prokaryotes **(A)** and fungi **(B)**. Plots show the ordinations for individuals colored according to dung beetle species **(A)** and dung type **(B)**. The stress (a measure of goodness of fit) was 0.187 for prokaryotes and 0.206 for fungi. (OFT, *O. fracticornis*; OMD, *O. medius*; OVT, *O. verticicornis*).

The ordination plot of the 90 individuals according to the prokaryotic component of their microbiota showed a clearer separation of points in the nMDS space when we grouped individuals according to species (**Figure 1A**) instead of dung type (**Supplementary Figure S4A**). The envfit analysis confirmed this result as species showed the highest R^2^ value (R^2^ = 35.7%; p = 0.001). Dung type and sex accounted for 15% (p = 0.001) and 10.1% (p = 0.001), respectively. The R^2^ value indicated that species was the strongest factor in differentiating individuals, but at the same time, the significant p-value also found for dung type and sex showed that these two other factors also contribute to differentiating individuals even if to a smaller extent.

Conversely, for fungi, the ordination plot of the 90 individuals showed a clearer distribution of the points in the nMDS space when we grouped the individuals according to dung type (**Figure 1B**) instead of species (**Supplementary Figure S4B**). R^2^ values confirmed these results as we found that the dung type showed the highest R^2^ value (R^2^ = 30.4%; p = 0.001). Species and sex accounted for 5.2% (p = 0.051) and 1.3% (p = 0.282), respectively. Furthermore, in this case, species was only nearly significant, and sex was not significant, so these two factors do not seem to contribute to the differentiation of individuals. However, we observed a clear separation of individuals collected from cow dung and instead a general overlap between individuals collected from sheep and donkey dung.

**Figure 2:**
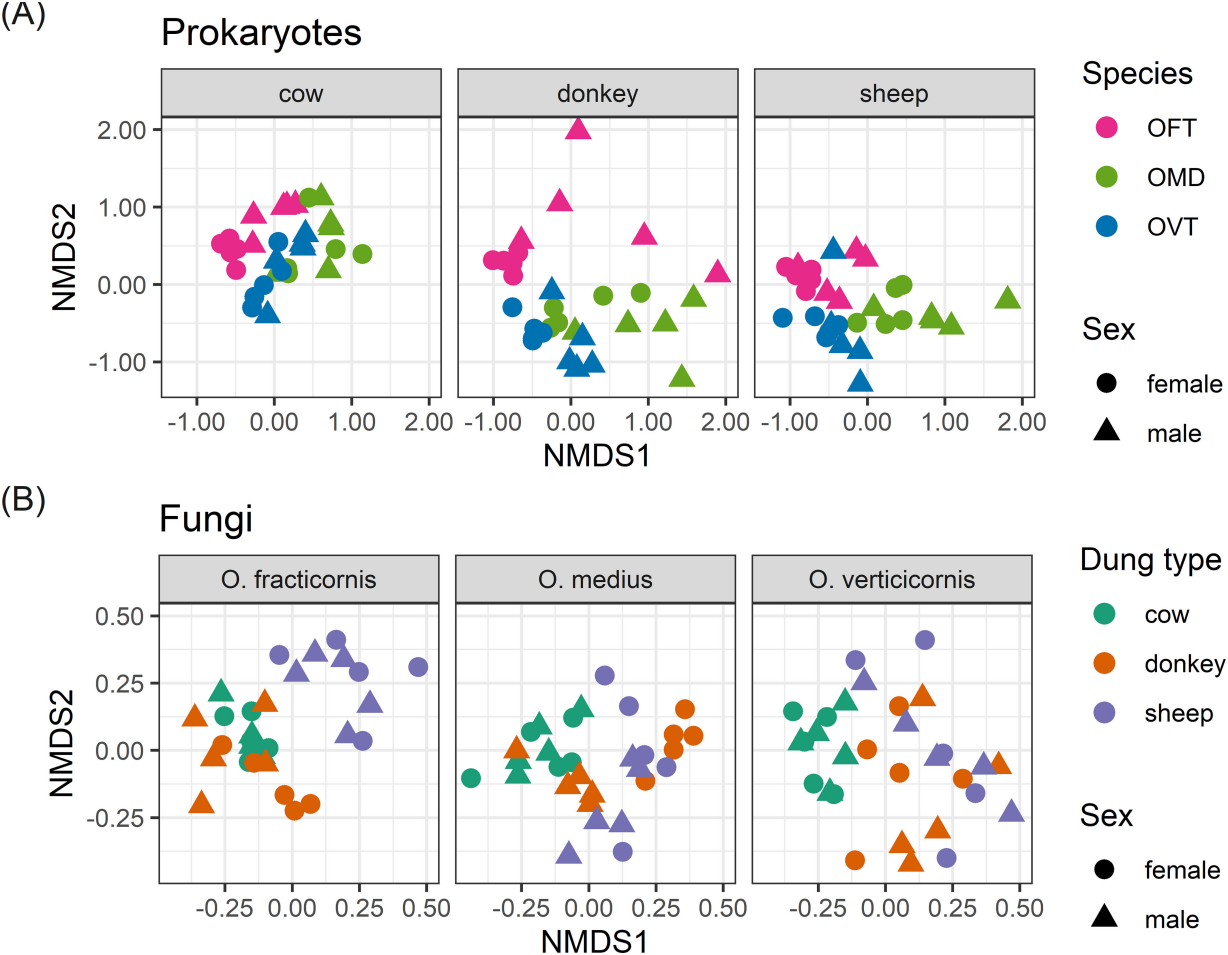
nMDS ordination plots for prokaryotes **(A)** separated by dung type in three different plots, and fungi **(B)** separated by species in three different plots. The stress (a measure of goodness of fit) was 0.187 for prokaryotes and 0.206 for fungi. (OFT, *O. fracticornis*; OMD, *O. medius*; OVT, *O. verticicornis*).

The ordinations in **Figure 1** showed good diversification between samples with regard to prokaryotes when we grouped individuals by species, and with regard to fungi when we grouped individuals by dung type. Thus, using the same nMDS results, the distinction between individuals was even greater when we considered the effect of the most important factor only in each of the two cases (**Figure 2**). When we separated the prokaryotes according to dung type, we noticed the effect of species in differentiating individuals. For example, OFT was well separated from the other species in all dung types (**Figure 2A**). In contrast, the distinction between OMD and OVT was less marked albeit still evident, especially in sheep dung, where the distinction between the three species was particularly good despite the presence of an OVT outlier. The effect of sex was mainly visible between individuals collected in donkey dung for all three species. We also observed an effect of sex in OFT in cow dung. However, the effect of sex in differentiating individuals was not appreciable in the other species or dung types. Considering, once more, the R^2^ values, sex was the factor with the lowest value.

With regard to fungi, when we separated the samples according to species, we noticed the effect of dung type in differentiating individuals. Mainly, we observed a clear separation between cow and sheep dung in all species (**Figure 2B**). Donkey dung samples, on the other hand, were always slightly mixed with the other dung types, especially in OMD where there was a large overlap between donkey dung and cow and sheep dung. For fungi, the effect of sex was even less evident than what was observed for prokaryotes. The only group with good separation between males and females was the OMD collected in donkey dung. R^2^ and p-values identified sex as a weak and non-significant factor.

### Exclusive and core ASVs

We evaluated the quantity of exclusive and core ASVs by considering both *i*) the number of ASVs and *ii*) the number of reads per ASV, as relative percentages (i.e. the relative abundance of exclusive and core ASVs).

**Figure 3:**
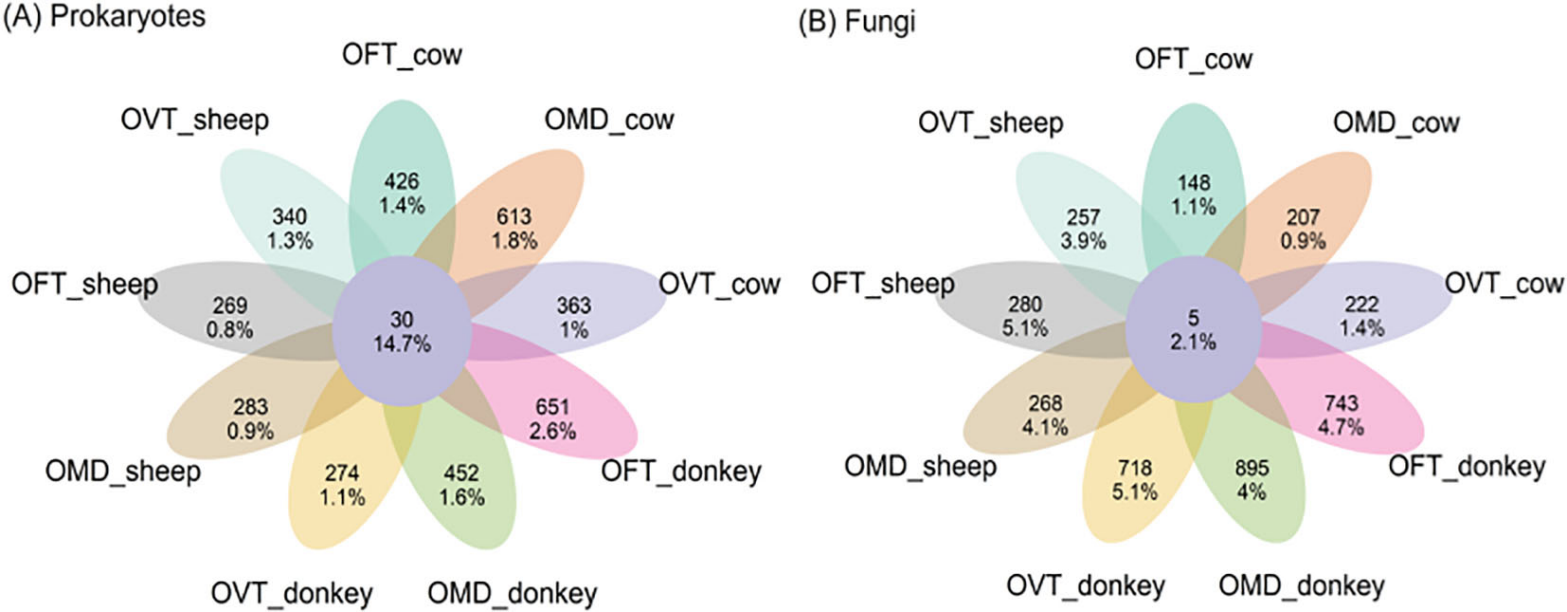
Flower plots presenting the relative abundances (percentages) of core and exclusive ASVs for prokaryotes **(A)** and fungi **(B)** in each species-dung type pair (OFT, *O. fracticornis*; OMD, *O. medius*; OVT, *O. verticicornis*). The numbers indicate the number of ASVs, whereas the percentages refer to the number of ASV reads, i.e. the relative abundances as percentages. In the center of each flower plot, the figures refer to the ASVs shared by all nine pairs, i.e. core ASVs, while in the petals the figures refer to those ASVs exclusive to each pair.

Overall, exclusive prokaryotic ASVs (i.e. those exclusive to each of the nine species-dung type pairs, **Figure 3**, see petals) comprised 78.8% of the 4659 detected ASVs and 12.5% of the 1193 detected reads. Exclusive fungal ASVs comprised 82.2% of the 4549 detected ASVs and 30.3% of the 1901 detected reads. The number of prokaryotic and fungal ASVs which were exclusive in each species-dung type pair were similar (on average 408 for prokaryotes, and 415 for fungi, 8.8% and 9.1%, respectively), but the ASV relative abundances were significantly lower in prokaryotes (on average 1.4%) than in fungi (3.4%). Core ASVs (i.e. those shared between all nine species-dung type pairs) were very few: 30 in prokaryotes and only 5 in fungi, corresponding to 0.6 and 0.1% of the detected ASVs, respectively; however, the percentage of core ASV abundances were higher with regard to their number, corresponding to 14.7% and 2.1%, respectively.

### Indicator ASVs

We detected very few indicator ASVs (i.e. those significantly associated with species, dung type or pairs) (**Table 1**), and ASVs associated with only one of the three dung beetle species were more numerous in prokaryotes than in fungi (on average 73.7 and 5.3, respectively). Vice-versa, ASVs associated with just one of the three dung types were more numerous in fungi than in prokaryotes (on average 45.7 and 24, respectively). We detected very few indicator ASVs across the nine species-dung type pairs in both prokaryotes and fungi, on average 7.4 and 5.7, respectively.

**Table 1:** Indicator ASVs associated with each group (i.e. dung type, species, or the nine pairs) visualized for both prokaryotes and fungi. .

Group	Number of associated ASVs (prokaryotes)	Number of associated ASVs (fungi)
cow	47	58
donkey	5	55
sheep	20	24
*O. fracticornis*	51	7
*O. medius*	70	8
*O. verticicornis*	100	1
OFT_cow	4	13
OFT_donkey	4	3
OFT_sheep	5	11
OMD_cow	6	5
OMD_donkey	10	9
OMD_sheep	13	2
OVT_cow	5	1
OVT_donkey	1	6
OVT_sheep	19	1

Only statistically significant ASVs (p < 0.05) are reported. (OFT, *O. fracticornis*; OMD, *O. medius*; OVT, *O. verticicornis*).

### Microbial taxonomic composition

**Figure 4:**
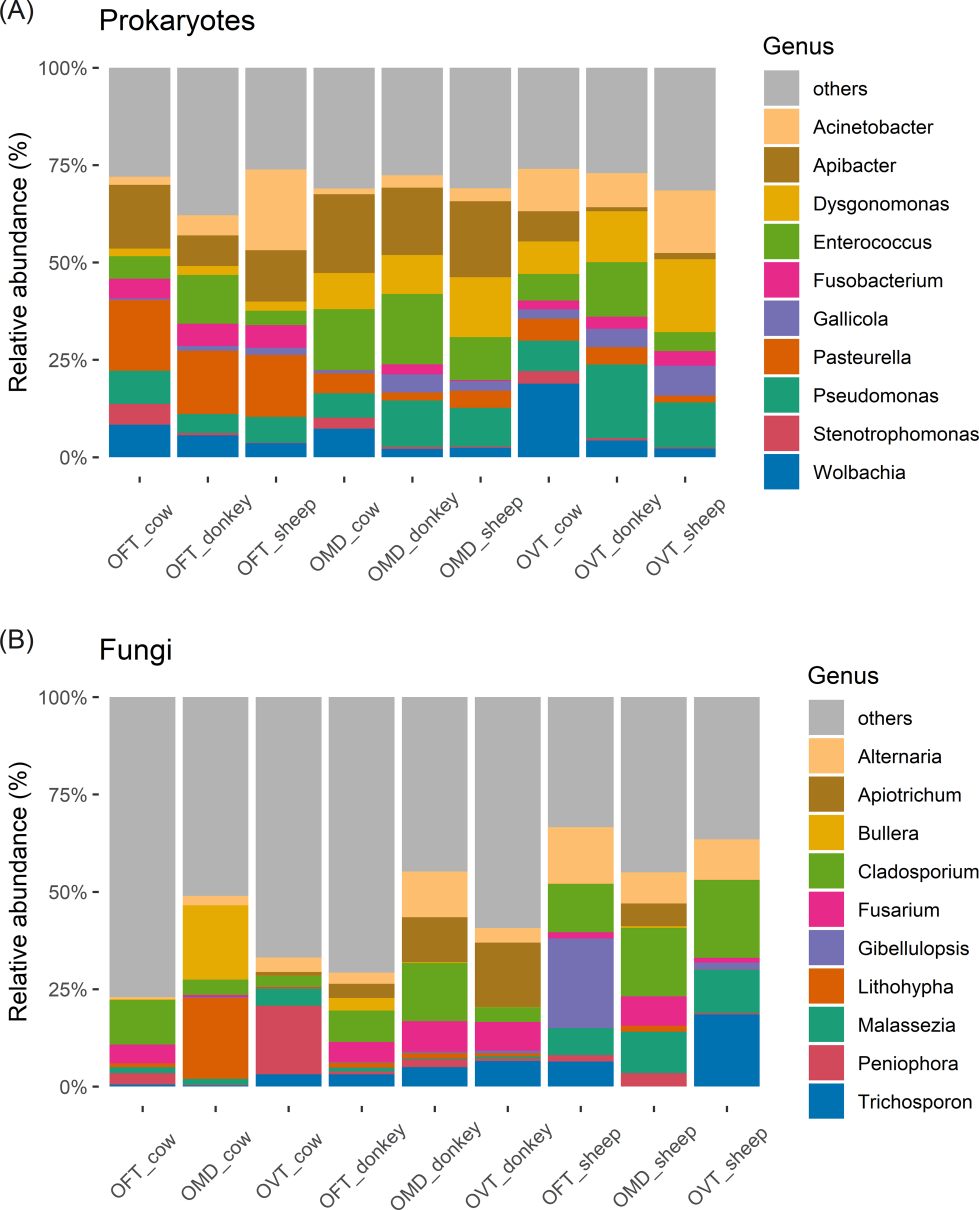
Stacked bar chart of the top ten most abundant microbial genera of prokaryotes **(A)** and fungi **(B)** in the nine species-dung type pairs. (OFT, *O. fracticornis*; OMD, *O. medius*; OVT, *O. ver*ticicornis).

Among the prokaryotes, Proteobacteria, Bacteroidota and Firmicutes accounted for more than 80% of the ASVs (**Supplementary Figure S5A**), while Archaea were barely detectable. At the genus level (**Figure 4A**), considering all samples, the top three genera in terms of abundance were *Apibacter*, *Enterococcus* and *Pseudomonas*. However, the taxonomic composition of each pair proved to be relatively constant regardless of the dung type, whilst varying considerably from one host species to another. In particular, the only significant difference between dung types was found for *Stenotrophomonas* (F_2,6_ = 20.6, p < 0.01), which was significantly more abundant in cow dung, whereas several genera showed significantly different abundances between the dung beetle species. For example, the relative abundances of *Pasteurella* varied between the three host-species (F_2,6_ = 64.478, p < 0.001), with higher values in OFT than in OMD (17% ± 1% vs 4% ± 2%; p < 0.001) or OVT (17% ± 1% vs 4% ± 2%; p < 0.001). At the same time, OFT hosted significantly fewer *Dysgonomonas* than OMD (2% ± 0% vs 11% ± 3%; p < 0.05) or OVT (2% ± 0% vs 13% ± 6%; p < 0.05). *Apibacter* was less abundant in OVT than in OFT (4% ± 4% vs 12% ± 4%; p < 0.05) or OMD (4% ± 4% vs 19% ± 2%; p < 0.01). *Acinetobacter* was observed in all samples, with no significant differences detected between species (F_2,6_ = 1.768, p = 0.249). Nevertheless, the highest percentage of abundance for *Acinetobacter* was observed in OFT collected from sheep dung (21%). The genus *Wolbachia* was found in all samples, with no significant differences between species (F_2,6_ = 0.497, p = 0.632); however, *Wolbachia* did account for a considerable percentage in OVT collected from cow dung (19%).

With regards to fungi, Ascomycota and Basidiomycota were the most abundant phyla, accounting for more than 95% of the ASVs (**Supplementary Figure S5B**). At the genus level (**Figure 4B**), considering all samples, the top three genera in terms of abundance were *Cladosporium*, *Alternaria*, and *Trichosporon*. Nevertheless, the taxonomic composition of each pair was relatively constant regardless of beetle species, whilst varying from one dung type to another. In particular, we found significant differences in the abundance of *Malassezia* in relation to dung type (F_2,6_ = 25.731, p < 0.01), with higher values in sheep dung than in cow dung (10% ± 2% vs 2% ± 2%; p < 0.01) and donkey dung (10% ± 2% vs 0% ± 1%; p < 0.01). Overall, while donkey dung and sheep dung samples showed a more constant internal set-up, we observed large internal differences in cow dung, even at the species level; for example, OFT was dominated by *Cladosporium* (11%), OMD showed a high abundance of *Lithophyla* and *Bullera* (21% and 19%, respectively), and OVT was dominated by *Peniophora* (18%). On the other hand, beetles collected from donkey dung exhibited high relative abundances of *Apiotrichum* and *Fusarium*, while *Cladosporium* and *Alternaria* were, on average, more abundant in beetles collected from sheep dung, although none of these differences were statistically significant (ANOVA).

For the sake of simplicity, all genera outside the ten most abundant have been pooled and labelled as ‘others’ in the bar charts (**Figure 4**). For prokaryotes, ‘other’ genera accounted for about 25% of the total microbial genera in each pair, whereas the percentages were much higher for fungi (on average more than 50%). For example, in the OFT-cow pair, ‘other’ genera accounted for more than 75%.

### Microbial functional composition

The functional composition of prokaryotes and fungi was relatively constant in all samples regardless of the species and type of dung consumed.

**Figure 5:**
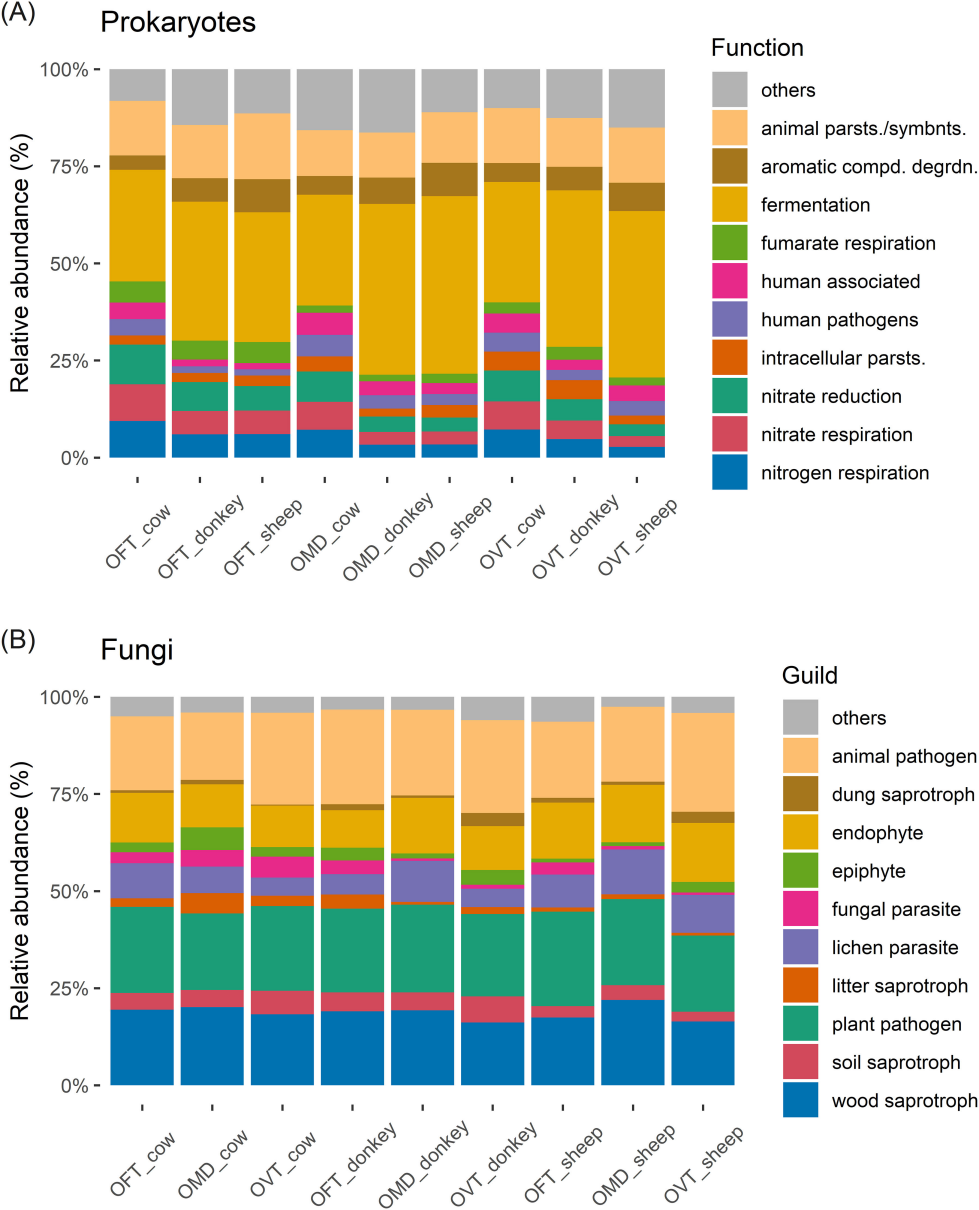
Stacked bar chart of the top ten most abundant prokaryotic functions **(A)** and fungal guilds **(B)** (OFT, *O. fracticornis*; OMD, *O. medius*; OVT, *O. verticicornis*; animal parsts./symbnts.: animal parasites or symbionts; aromatic compd. degrdn.: aromatic compound degradation; intracellular parsts.: intracellular parasites).

More than 95% of the detected prokaryotes had metabolisms based on aerobic or anaerobic chemoheterotrophy (**Supplementary Figure S6A**). The three host species showed approximately the same functional set-up (**Figure 5A**). The only prokaryotic function with a significantly different distribution in abundance between host species was fumarate respiration (F_2,6_ = 67.0, p < 0.001), which was more abundant in OFT than in OMD (5% ± 0% vs 2% ± 0%; p < 0.001) or OVT (5% ± 0% vs 3% ± 1%; p < 0.001). Fermentation, a typical carbon metabolic pathway, was the most common metabolic process in the prokaryotic microbiota, with an average of 37% ± 7% of reads, irrespective of the species considered. However, some differences in the abundances of fermentation function across dung types should be noted (F_2,6_ = 5.378, p < 0.05). These weak significant differences mainly seemed to be related to cow dung for which fermentation was relatively low, whereas the nitrogen-related metabolic pathways were more prevalent (i.e. nitrate respiration: F_2,6_ = 5.15, p < 0.05; nitrate reduction: F_2,6_ = 7.389, p < 0.05; nitrogen respiration: F_2,6_ = 5.15, p < 0.05). However, when examining the Tukey HDS results, all comparisons between dung types resulted non-significant, except for nitrate reduction, which was more abundant in beetles collected from cow dung than from sheep dung (9% ± 1% vs 4% ± 2%; p < 0.05). A relevant percentage in each pair consisted of prokaryotes identified as parasites or animal symbionts (on average 14% ± 2%, regardless of the species and dung type considered).

Regarding fungi, from a broader perspective, the most common trophic modes were saprotroph, followed by pathotroph and symbiotroph (**Supplementary Figure S6B**). Plant pathogens, animal pathogens and wood saprotrophs were the most common guild in the fungal microbiota, averaging 22% ± 1%, 22% ± 3% and 18% ± 2%, respectively, regardless of the host species and dung type considered (**Figure 5B**). No significant differences were found in relation to dung type or host species, with the exception of wood saprotrophic fungi (F_2,6_ = 6.067, p < 0.05), which were slightly more abundant in OMD than in OVT (20% ± 2% vs 17% ± 1%, respectively; p < 0.05). The relative abundance of the dung saprotroph guild was equal in all pairs (1% ± 1% on average; no significant differences in relation to dung type or host species).

## Discussion

Our main objective was to reveal whether phylogeny (i.e. dung beetle species) or diet (i.e. the dung type consumed) was the predominant factor that shaped the differences in the prokaryotic and fungal gut microbiota in adult individuals of three species *Onthophagus* beetles. Our second aim was to compare taxonomic and functional variability to check for functional similarities between individuals. To achieve these goals, we performed molecular analyses on the beetles’ gut microbiota and analyzed the alpha and beta diversities, core ASVs, indicator ASVs, taxonomy and functionality of the considered microbes.

The alpha diversity analyses revealed the diversity parameters (Shannon Index or Faith PD index) to vary according to diet, species and sex, but they did not detect any clear differences between the diversity patterns of prokaryotes and fungi. Conversely, the results of all other analyses concerning ASV identity consistently revealed clear differences in the patterns of ASV composition for both prokaryotes and fungi. Indeed, beta diversity analysis (i.e. the nMDS ordination) for prokaryotes revealed the individuals to cluster according to the three *Onthophagus* species, whereas the analysis for fungi showed a clustering according to dung type. Indicator ASVs related to each of the three dung beetle species were much more numerous in prokaryotes than in fungi, while ASVs related to the three dung types were much more numerous in fungi than in prokaryotes. Finally, the prokaryotic taxonomic composition varied from one dung beetle species to another (but was constant within each species regardless of the dung type), while the fungal one tended to vary from one dung type to another (being relatively constant within each type of dung regardless of the species). These results suggest that microbial composition analyses are more effective at highlighting patterns of variation dependent on phylogeny and diet. They also suggest that the composition of the dung beetle gut microbiota reflects two divergent trends: that the prokaryotic component mostly depends on the identity of the host species, while the fungal component greatly depends on diet (i.e. the type of dung just ingested by the host). The phylogeny of the host and its diet are therefore key factors driving microbial composition, as confirmed by other studies on beetle species [e.g. (21, 53)]. By consequence, we can hypothesize that most prokaryotes of dung beetle microbiota are definitive symbionts, whereas many fungi are transient symbionts. It has been shown that certain lineages of bacteria that are beneficial for host nutrition can be transmitted from mother to offspring, creating a kind of species-specific microbiota. This phenomenon is particularly present in *Onthophagus* species, given their approach to parental care and the vertical transmission of the microbiota via pedestals (14, 16, 20, 54). Fungi, on the other hand, being more dependent on the host beetle’s diet can generally be referred to as transient symbionts. Similar results have been reported for the gut microbiota in certain caterpillar species, where bacteria were indicated to be a core component of the microbiota, and fungi represented a more transient component (55).

Another interesting result testifying to the differences between the individuals tested was the scarcity of a core microbiota and, in a complementary way, the abundance of exclusive ASV. As pointed out in the flower plots, the ASVs shared between all nine pairs were in fact very few, representing less than 1% of the total number of ASVs, whereas exclusive ASVs (i.e. those found in one pair only), were more numerous, accounting for approximately 80% of the total number of ASVs for both prokaryotes and fungi. These results are consistent with previous findings for *T. pyrenaeus* and *P. striatum*, which showed that the core microbial sequences, both bacterial and fungal, were invariably less numerous than those that were exclusive to the individuals (19, 23). Nevertheless, there is the possibility that this large level of exclusivity is, however, of little biological significance. For instance, if a certain ASV is detected only once in the total dataset, it is obvious that it is ‘statistically exclusive’ to the pair in which it was found, but it is also unlikely to be associated with any biological importance. ‘Biological exclusivity’, on the other hand, assumes that all the many similar ASVs are exclusive to a species when it feeds on a certain type of dung. From this point of view, it is crucial to place due emphasis on the findings related to indicator ASVs, which showed that very few ASVs were truly associated with a particular species or dung type or species-dung type pair.

Many of the prokaryotic and fungal gut microbes found during our analyses have been indicated by previous studies as efficient providers of functions that benefit dung beetles. Here, we found Proteobacteria and Firmicutes to be among the most abundant phyla in the prokaryotic microbiota, consistent with previous studies (11, 15, 19). Firmicutes, associated with fiber-rich diets (11, 56), may help to break down complex polysaccharides such as cellulose and hemicellulose. The genus *Pseudomonas* seems to be highly beneficial in amino acid metabolism, nitrogen fixation and lignocellulose degradation (6, 10, 22). *Acinetobacter*, also identified as an abundant genus in previous research (11, 19, 21, 22), is known to decompose organic material such as dung or carcasses, making it a beneficial genus for dung beetles. The genus *Dysgonomonas* appears to be very useful for the synthesis of antifungal compounds, in particular, against entomopathogenic fungi such as *Metarhizium* spp (57). Furthermore, *Dysgonomonas*, known nitrogen fixers (20, 58), are also found in fungus-growing termites, where they possibly hydrolyze cellulose (59), and may have similar functions in beetles; for example, in the degradation pathway of lignocellulosic biomass and in providing easily metabolized substrates for host ingestion (10, 20, 23). Some pathogenic bacteria have also been found in dung beetles such as *Pasteurella*, known zoonotic pathogens, most probably originating from domestic animals (cows, sheep or horses) as shown in previous studies (21). *Wolbachia*, on the other hand, is a bacterium that infects many insect species and may even alter the microbiota, although it has been observed as a common member of the dung beetle microbiota (21, 22, 56, 60). *Wolbachia* infections have different effects on host insects, ranging from beneficial i.e., nutrient supplementation and protection from viruses (57, 61) to deleterious functions i.e., feminization and the killing of males (57).

Regarding the fungi present in the microbiota, the Trichosporonaceae taxa, abundantly present in many individuals, have been indicated to be capable of assimilating or degrading lignocellulose (62). Genera of this family, such as *Apiotrichum* and *Trichosporon*, have also been observed to be predominant in other dung beetle species (11, 19, 62). For example, in the dung beetle *Copris acutidens*, the genus *Apiotrichum* was found in both larvae and adults, indicating the possibility that this symbiotic fungus may be transmitted to the larval gut by the brood balls (62). The genus *Cladosporium* was found to have cellulolytic and xylanolytic properties towards various aquatic insects (63), supporting the hypothesis that these fungi can improve the digestibility of plant material by insects, and in our case the digestibility of residual plant material contained in dung.

The large number of functions potentially covered by the prokaryotes and fungi found in the gut of the three species of dung beetles confirms the biological relevance of these microorganisms. They ensure the health of the host organisms and are at the same time responsible for the degradation processes of the dung deposited by wild and domestic ungulates in pastures. The use of functional prediction tools such as FAPROTAX (51) and FUNGuild (52) relies on the assumption that taxonomy can be used as a proxy for function. Despite some limitations, such as dependence on literature-based databases that are not frequently updated and predictions not derived from actual genetic content (64), these tools offer a cost-effective means to gain preliminary insight into the putative functional potential of microbial communities, particularly when metagenomic or metatranscriptomic data are not available. Future work integrating multi-omics approaches will help to validate and refine these functional predictions.

Another objective of this research was to compare the taxonomic and functional variability of gut microbiota. As also highlighted in previous work (19, 23, 33), it might be more commonplace and meaningful to look for a functional rather than a taxonomic core. Indeed, this also seemed to be the case in the present study. Our results showed that the large taxonomic differences between the different groups did not translate into any relevant functional diversity. Indeed, in relation to both prokaryotes and fungi, all the groups considered presented roughly the same pattern of functions, highlighting the contrast between the strong taxonomic dissimilarity and the wide similarity in functional composition. Indeed, it appears that the microbiota has a certain resilience to environmental and/or diet-related disturbances, which ensures the host the maintenance of essential metabolic functions despite recurrent disturbances (65). This means that although the microbes present in the gut microbiota may change even substantially, the functions potentially expressed remain essentially the same. This concept is grounded in what is referred to in the literature as functional redundancy (66, 67). Thus, the functional redundancy hypothesis, which was initially tested on other organisms, also seems applicable to dung beetles, although further analysis would be required. In turn, this preservation of microbial functionality, despite differences in microbial taxonomy, may contribute to ensuring the ability of the dung beetle to successfully feed, survive and reproduce, and by consequence the continual provision of ecological services to the ecosystem.

## Conclusions

To the best of our knowledge, this is the first work to show how the cause of prokaryotic and fungal diversity in the dung beetle gut microbiota is different. We found that the prokaryotic component of the microbiota varies according to the host species, and may be different even in phylogenetically very close species. *Vice versa*, we show that fungi derive, at least in part, from the host’s diet (i.e. through different types of dung). This difference should be carefully considered in studies of the gut microbiota of dung beetles because the evolutionary and ecological ‘logic’ affecting the two groups are probably very different. Future studies are needed to further confirm these results, potentially involving a larger number of species, even from different genera. At the same time, it is necessary to consider that, although the functional role of the microbiota is usually ascribed to bacteria, the different fungal guilds also provide a significant functional contribution.

Studying wild individuals allowed us to investigate conditions that are as close to natural conditions as possible. This is an objective strength of the present work since research on the microbiota of individuals raised in the laboratory, although useful for discovering fundamental processes such as those linked to the vertical transmission of the microbiota (68), cannot provide data on the variability in microbiota composition in wild individuals. Thus, sampling individuals from the wild allowed us to fully appreciate the great variability in the gut microbiota of these beetles, and this finding sets the scene for a future challenge: to isolate, cultivate and identify the bacterial and fungal taxa associated with the digestive tract of *Onthophagus* spp. to reveal the functional dimension of their gut microbiota. Indeed, the integration of culture-independent and culture-dependent approaches is a key strategy for the in-depth characterization of the functional diversity of this species and its ecological significance across various environments. In addition, the removal of environmental ASVs from those present in the gut and the purging of the beetle’s gut for at least 24 hours before dissection made it possible to consider more rigorously only those sequences constituting the gut microbiota. In this way, contamination with microbes possibly derived from soil or ingested food can be limited.

Finally, the results of this study also provide useful analytical indications. Precisely, our findings suggest analyses of microbial composition to be more effective than analyses of alpha diversity for identifying patterns of variation dependent on phylogeny and diet.

## Data Availability

The raw data used to generate the dataset for this study can be found in the NCBI Sequence Read Archive (SRA) under accession no. PRJNA1298534 and PRJNA1298152 for prokaryotic and fungal communities respectively.
