# Supplementary material for: Gut microbiota variability in dung beetles: prokaryotes vary according to the phylogeny of the host species while fungi vary according to the diet
Source: Front Insect Sci. 2025 Aug 20;5:1639013. doi: 10.3389/finsc.2025.1639013 (PMC12405213; doi:10.3389/finsc.2025.1639013)
Supplement: Supplementary file 1 [file DataSheet1.pdf]

Supplementary File 1

Assessment of the phylogenetic relationships of the three dung beetle species

To compare phylogenetic relationships between the three target species (i.e. *O. fracticornis, O. medius, O. verticicornis*) and between the target species and other *Onthophagus* species (i.e. *O. taurus*, *O. illyricus*, *O. grossepunctatus* and *O. lemur and O. furcatus*) collected in Mocchie (Piedmont, Italy) we selected four individuals (two males and two females) per species, and removed a foreleg from each individual for molecular analysis (Table S1).

Each limb was preserved in absolute ethanol and stored at 4°C. DNA was extracted using a slightly modified CTAB method (1,2), as described in Natta *et al.* (2024) (3). We applied a DNA barcoding approach to investigate the phylogenetic relationships between the three species using mitochondrial sequences of the cytochrome oxidase subunit I (COI) and the internal transcribed spacer region 2 (ITS2) as molecular targets. The COI gene was amplified using primers LCO1490 (5'-CHACWAAYCATAAAGATATYGG) and HCO2198 (5'-AWACTTCVGGRTGVCCAAARAATCA) (4,5), while the ITS2 gene was amplified using the primers ITS2-F (5'-GGGTCGATGAAGAACGCAGC) and ITS2-R (5'-ATATGCTTAAATTCAGCGGG) (6). PCR were run in 25 µl using 10 µmol of each primer, 5 µmol of FIREPol® Master Mix (Carlo Erba Reagents) and 20 ng of DNA. The PCR conditions were: 5 min at 95°C, followed by 35 cycles of 30 s of denaturation at 95°C, 45 s of annealing at 50°C and 2.5 min of extension at 65°C, with a final single extra extension step of 5 min at 65°C. PCR products were visualised using 1% agarose gel with 2 µl of ethidium bromide and purified using Wizard® SV gel and PCR Clean-Up System (Promega). We then quantified the DNA with QUBIT (Invitrogen) and sent the purified samples to Genechron (Rome, Italy) for sequencing.

We examined the phylogenetic relationships between the species collected in the Mocchie area using both COI and ITS sequences together in MAFFT v7, a multiple sequence alignment (MSA) program (7,8), using the MAFFT online resources (https://mafft.cbrc.jp/alignment/server/). Since ITS sequences are not usually regarded as reliable for assessing interspecific diversity within Coleoptera, we did not analyse them beforehand using ASAP (9). We aligned the matrix sequences in MAFFT (*auto* strategy) and built a NJ phylogenetic tree. Furthermore, to confirm the former results, the alignment produced in MAFFT was also used to produce a maximum likelihood tree in MEGA12 (best model = T92+G+I selecting the adaptative bootstrap option to test the phylogeny, (10)) and a Bayesian inference tree (MrBayes v3.2, (11)) with the model settings: data = not partitioned; Nst = 1; covariation = no; rates = equal; N of generation = 1,000,000; number of chains = 2; burnin fraction = 0.25%. In the former analyses, individuals of *Euoniticellus fulvus*, also collected in Mocchie, were used as an outgroup to root the calculated trees.

Table S1 . List of the COI sequences used in the phylogenetic analyses, with the accession codes in BOLD..

No	species	code BOLD	Locality
1	*Euoniticellus fulvus*	UTOCP021-25	Italy, Piedmont, Susa Valley, Mocchie
2	*Euoniticellus fulvus*	UTOCP020-25	Italy, Piedmont, Susa Valley, Mocchie
3	*Onthophagus fracticornis*	UTOCP011-25	Italy, Piedmont, Susa Valley, Mocchie
4	*Onthophagus fracticornis*	UTOCP010-25	Italy, Piedmont, Susa Valley, Mocchie
5	*Onthophagus fracticornis*	UTOCP012-25	Italy, Piedmont, Susa Valley, Mocchie
6	*Onthophagus furcatus*	UTOCP025-25	Italy, Piedmont, Susa Valley, Mocchie
7	*Onthophagus furcatus*	UTOCP023-25	Italy, Piedmont, Susa Valley, Mocchie
8	*Onthophagus furcatus*	UTOCP024-25	Italy, Piedmont, Susa Valley, Mocchie
9	*Onthophagus furcatus*	UTOCP022-25	Italy, Piedmont, Susa Valley, Mocchie
10	*Onthophagus grossepunctatus*	UTOCP026-25	Italy, Piedmont, Susa Valley, Mocchie
11	*Onthophagus grossepunctatus*	UTOCP027-25	Italy, Piedmont, Susa Valley, Mocchie
12	*Onthophagus illyricus*	UTOCP030-25	Italy, Piedmont, Susa Valley, Mocchie
13	*Onthophagus illyricus*	UTOCP031-25	Italy, Piedmont, Susa Valley, Mocchie
14	*Onthophagus illyricus*	UTOCP028-25	Italy, Piedmont, Susa Valley, Mocchie
15	*Onthophagus illyricus*	UTOCP029-25	Italy, Piedmont, Susa Valley, Mocchie
16	*Onthophagus lemur*	UTOCP036-25	Italy, Piedmont, Susa Valley, Mocchie
17	*Onthophagus lemur*	UTOCP037-25	Italy, Piedmont, Susa Valley, Mocchie
18	*Onthophagus medius*	UTOCP015-25	Italy, Piedmont, Susa Valley, Mocchie
19	*Onthophagus medius*	UTOCP016-25	Italy, Piedmont, Susa Valley, Mocchie
20	*Onthophagus medius*	UTOCP013-25	Italy, Piedmont, Susa Valley, Mocchie
21	*Onthophagus medius*	UTOCP014-25	Italy, Piedmont, Susa Valley, Mocchie
22	*Onthophagus taurus*	UTOCP033-25	Italy, Piedmont, Susa Valley, Mocchie
23	*Onthophagus taurus*	UTOCP034-25	Italy, Piedmont, Susa Valley, Mocchie
24	*Onthophagus taurus*	UTOCP032-25	Italy, Piedmont, Susa Valley, Mocchie
25	*Onthophagus verticicornis*	UTOCP018-25	Italy, Piedmont, Susa Valley, Mocchie
26	*Onthophagus verticicornis*	UTOCP019-25	Italy, Piedmont, Susa Valley, Mocchie
27	*Onthophagus verticicornis*	UTOCP017-25	Italy, Piedmont, Susa Valley, Mocchie

Phylogenetic relationships of the three dung beetle species

COI sequences confirmed that the three target species (*O. fracticornis*, *O. medius* and *O. verticicornis*) were correctly identified through the use of external morphological traits. Both the maximum likelihood tree (Fig. S1) and the Bayesian inference analysis consensus tree (Fig. S2) confirmed that all the collected individuals of the genus *Onthophagus* were clustered into 3 subgenera, i.e. *Furconthophagus*, *Onthophagus s.str*. and *Palaeonthophagus*. All three target species fell into the same cluster, namely, the subgenus *Palaeonthophagus*. The clade corresponding to the *Paleonthophagus* subgenus also included *O. grossepunctatus* and *O. lemur*, the latter being more closely related among themselves than the three target species.

The phylogenetic relationships between these three target *Onthophagus* species were confirmed by both analyses. However, O. *fracticornis* and *O. verticicornis* were more strictly related to each other than to *O. medius*. Also, the Bayesian analysis confirmed the closer relationships between the three species of the dataset, with a clade credibility value equal to 100% for each of the species included in the analysis.

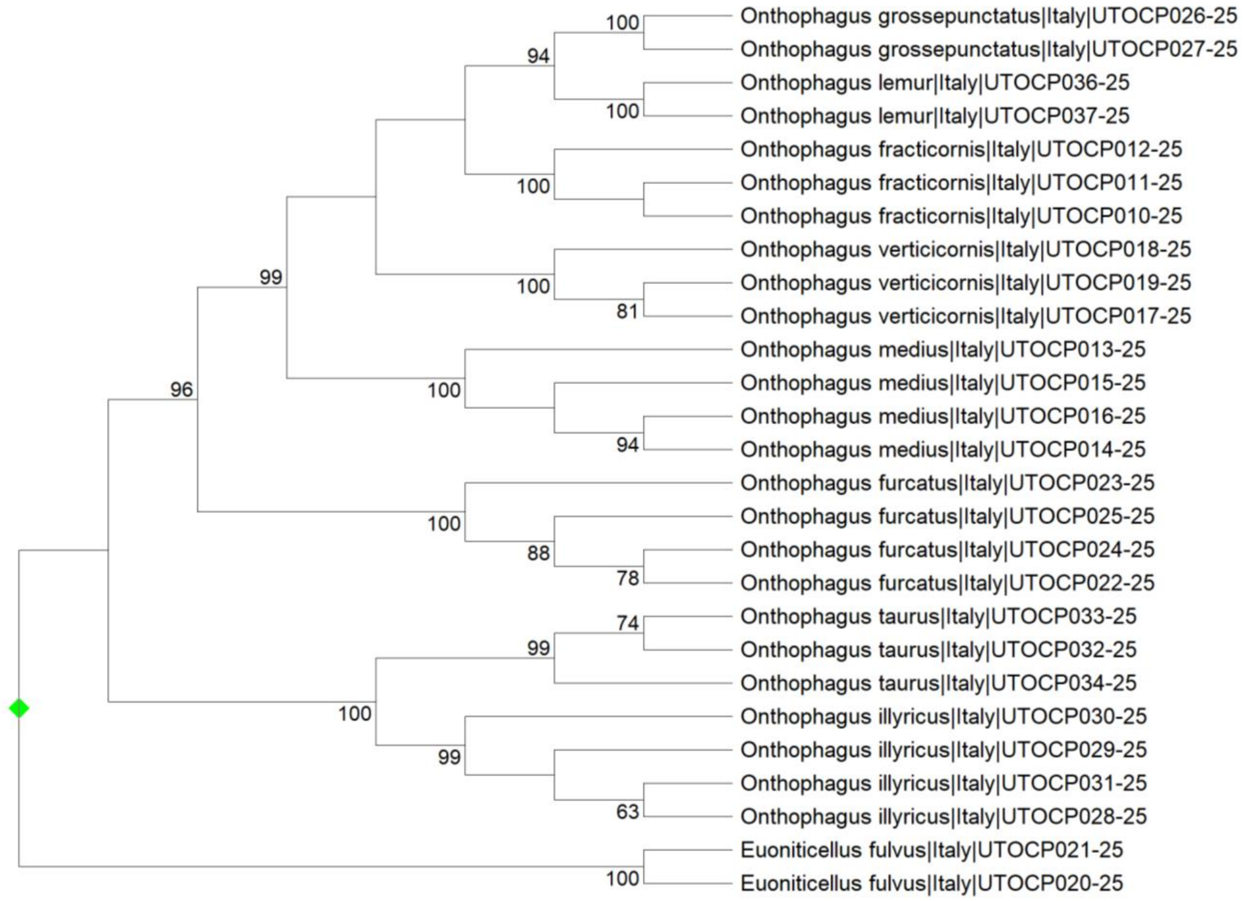

Figure S1. Maximum likelihood tree for the *Onthophagus* species collected in Mocchie. *Euoniticellus fulvus* from Mocchie was used as an outgroup to root the calculated tree. The tree (log likelihood = -10,074.17) shows the percentage of replicate trees (values>51%) in which the taxa clustered together next to the branches. The three *Onthophagus* subgenera (i.e., *Paleonthophagus*, *Onthophagus s.str*. and *Furconthophagus* constituted three distinct clades.

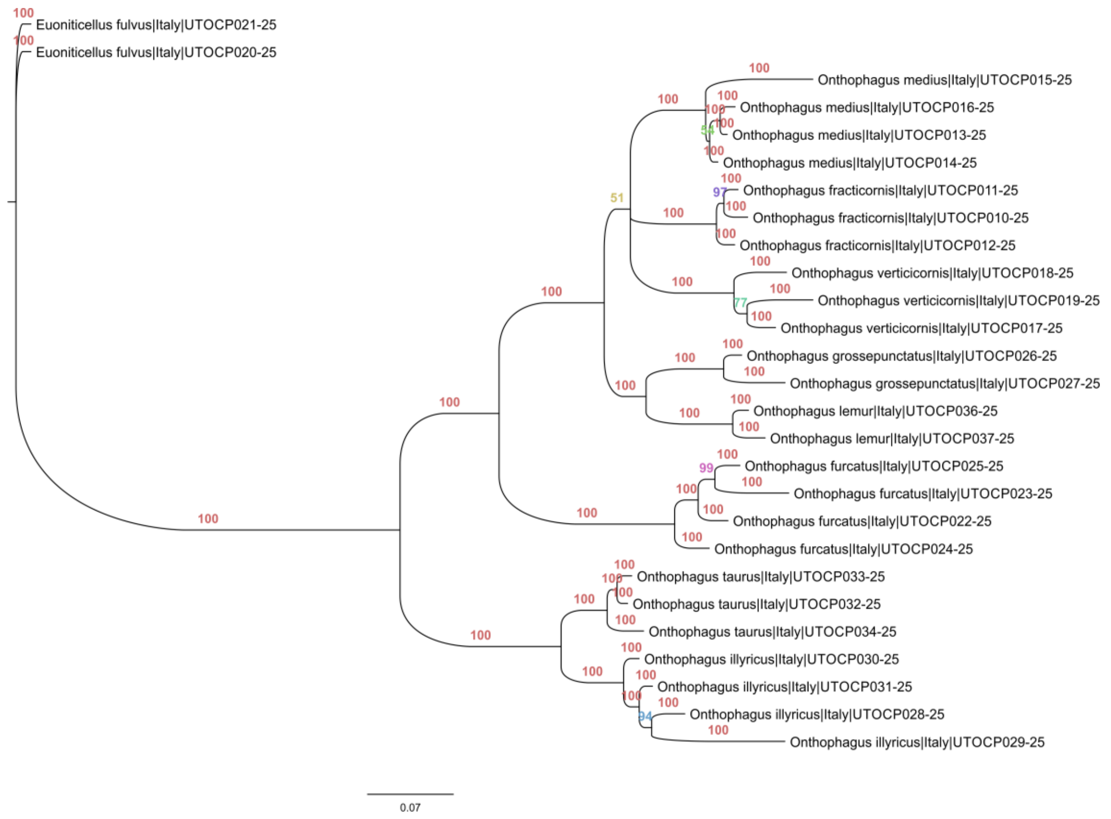

Figure S2. Bayesian inference analysis consensus tree for the *Onthophagus* species collected in Mocchie with the probability percent values of each node. *Euoniticellus fulvus* from Mocchie was used as an outgroup to root the calculated tree. The Bayesian analysis furnished higher percent values of probability for each clade than the ML tree.

References

1. Rogers SO, Bendich AJ. Extraction of DNA from milligram amounts of fresh, herbarium and mummified plant tissues. *Plant Mol Biol* (1985) 5:69–76. doi: 10.1007/BF00020088

2. Doyle J, Doyle JL. Isolation of plant DNA from fresh tissue. *Focus* (1990) 12:13–15.

3. Natta G, Voyron S, Lumini E, Laini A, Santovito A, Roggero A, Palestrini C, Rolando A. DNA metabarcoding of gut microbiota reveals considerable taxonomic differences among wild individuals of the dung beetle *Trypocopris pyrenaeus* (Coleoptera: Geotrupidae). *Eur J Entomol* (2024) 121:40–53. doi: 10.14411/eje.2024.007

4. Folmer O, Black M, Hoeh W, Lutz R, Vrijenhoek R. DNA primers for amplification of mitochondrial cytochrome c oxidase subunit I from diverse metazoan invertebrates. *Mol Mar Biol Biotechnol* (1994) 3:294–299.

5. Astrin J, Stüben P, Zfmk A, Forschungsmuseum Z, Koenig A. Phylogeny in cryptic weevils: molecules, morphology and new genera of western Palaearctic Cryptorhynchinae (Coleoptera: Curculionidae). *Invertebr Syst* (2008) 22: doi: 10.1071/IS07057

6. Navajas M, Lagnel J, Gutierrez J, Boursot P. Species-wide homogeneity of nuclear ribosomal ITS2 sequences in the spider mite Tetranychus urticae contrasts with extensive mitochondrial COI polymorphism. *Heredity* (1998) 80:742–752. doi: 10.1046/j.1365-2540.1998.00349.x

7. Kuraku S, Zmasek CM, Nishimura O, Katoh K. aLeaves facilitates on-demand exploration of metazoan gene family trees on MAFFT sequence alignment server with enhanced interactivity. *Nucleic Acids Res* (2013) 41:22–28. doi: 10.1093/nar/gkt389

8. Katoh K, Rozewicki J, Yamada KD. MAFFT online service: multiple sequence alignment, interactive sequence choice and visualization. *Brief Bioinform* (2019) 20:1160–1166. doi: 10.1093/bib/bbx108

9. Puillandre N, Brouillet S, Achaz G. ASAP: assemble species by automatic partitioning. *Mol Ecol Resour* (2021) 21:609–620. doi: 10.1111/1755-0998.13281

10. Tamura K, Stecher G, Kumar S. MEGA11: Molecular Evolutionary Genetics Analysis Version 11. *Mol Biol Evol* (2021) 38:3022–3027. doi: 10.1093/molbev/msab120

11. Ronquist F, Teslenko M, van der Mark P, Ayres DL, Darling A, Höhna S, Larget B, Liu L, Suchard MA, Huelsenbeck JP. MrBayes 3.2: efficient Bayesian phylogenetic inference and model choice across a large model space. *Syst Biol* (2012) 61:539–542. doi: 10.1093/sysbio/sys029
