# Supplementary material for: Gut microbiota variability in dung beetles: prokaryotes vary according to the phylogeny of the host species while fungi vary according to the diet
Source: Front Insect Sci. 2025 Aug 20;5:1639013. doi: 10.3389/finsc.2025.1639013 (PMC12405213; doi:10.3389/finsc.2025.1639013)
Supplement: Supplementary file 2 [file DataSheet2.pdf]

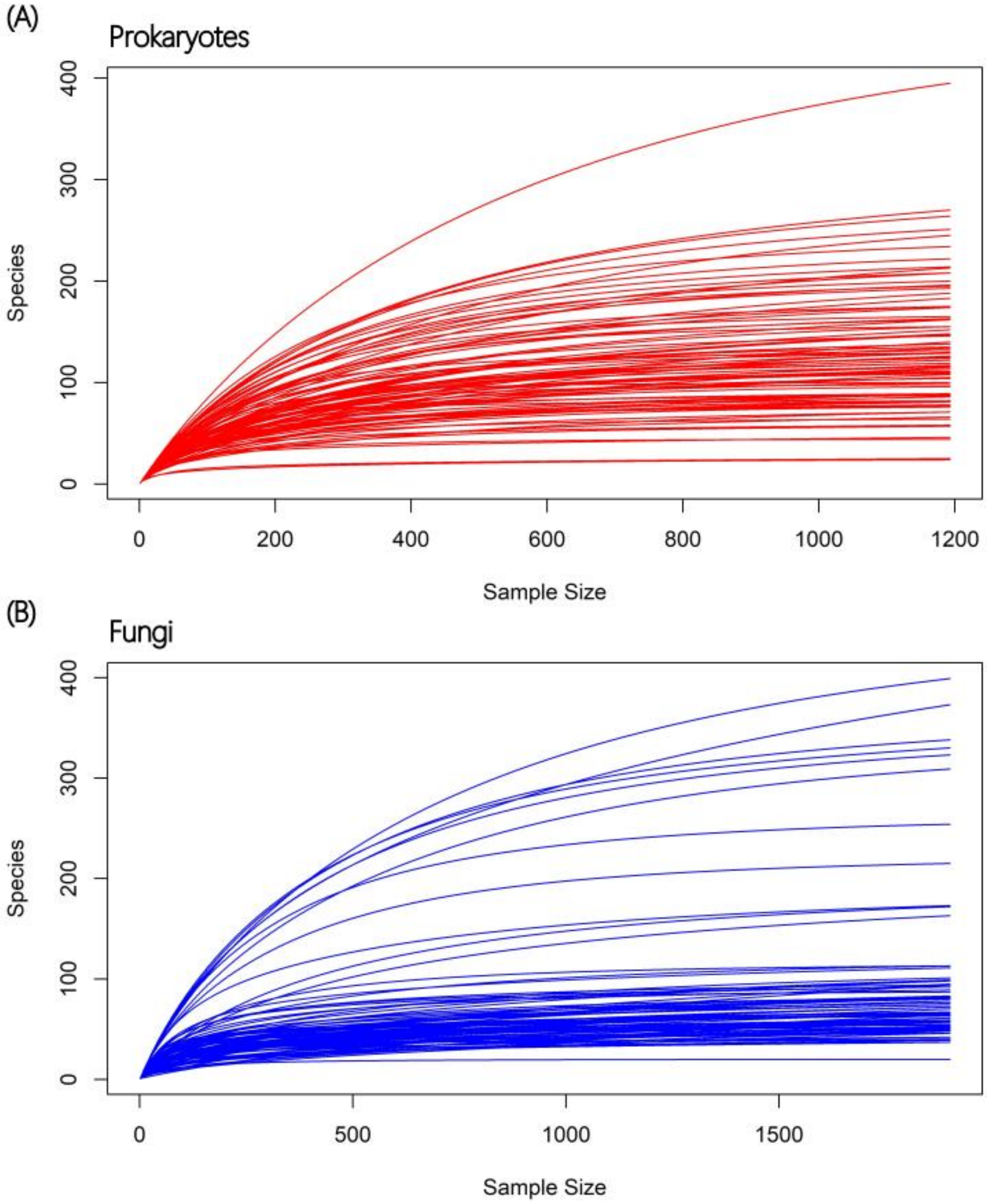

Figure S3. Rarefaction curves for prokaryotes (A) and fungi (B). The y-axis shows the ASV number found for each individual in relation to the number of reads (x-axis).

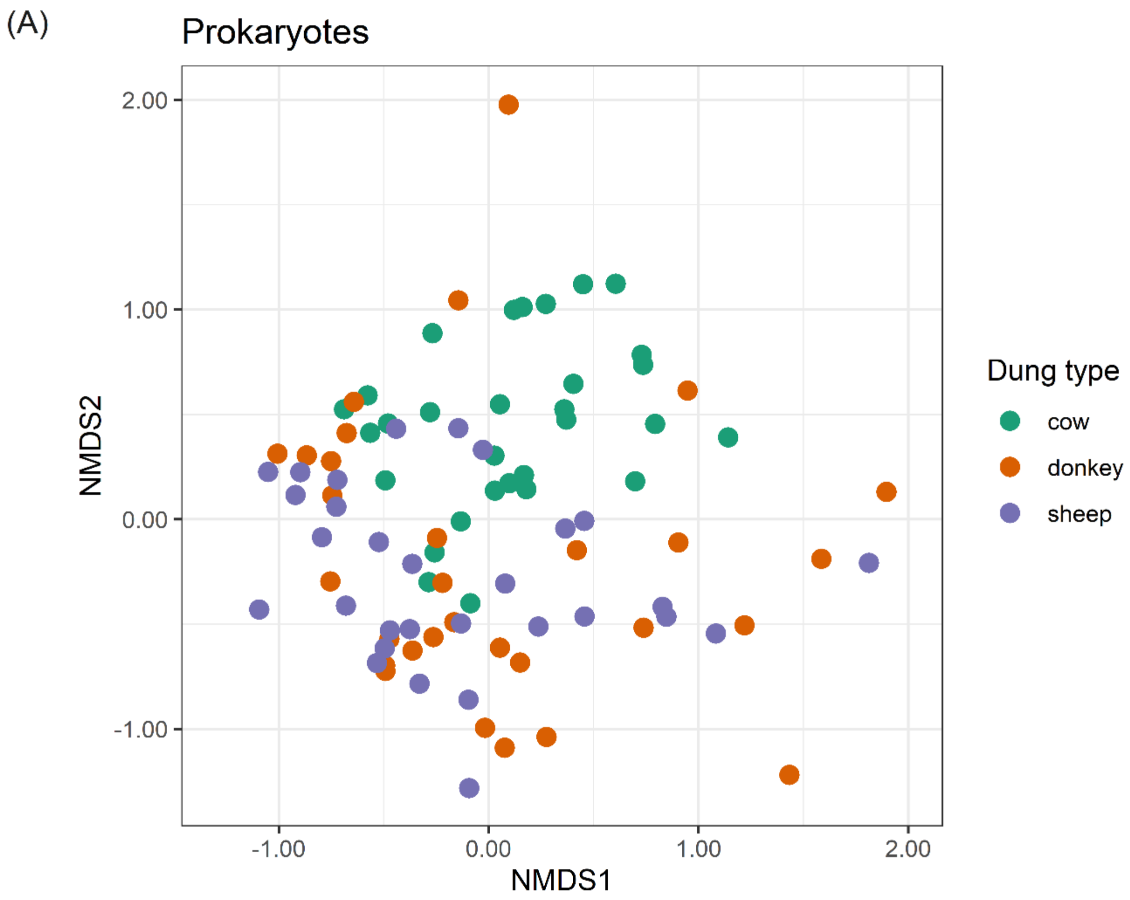

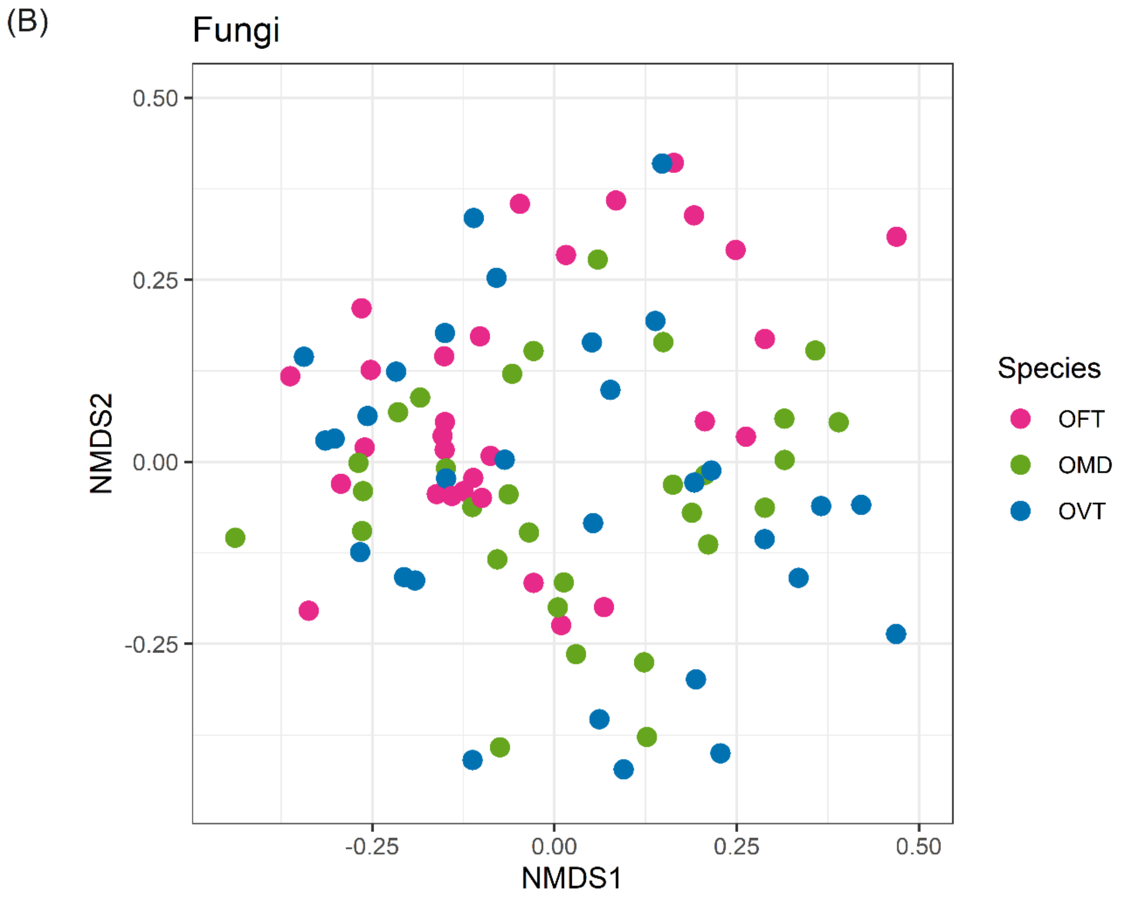

Figure S4. Results of a nMDS analysis for prokaryotes (A) and fungi (B). Plots show grouping by dung type (A) and dung beetle species (B). Stress (a measure of goodness of fit) is 0.187 for prokaryotes, and 0.206 for fungi. (OFT: *O. fracticornis*; OMD: *O. medius*; OVT: *O. verticicornis*).

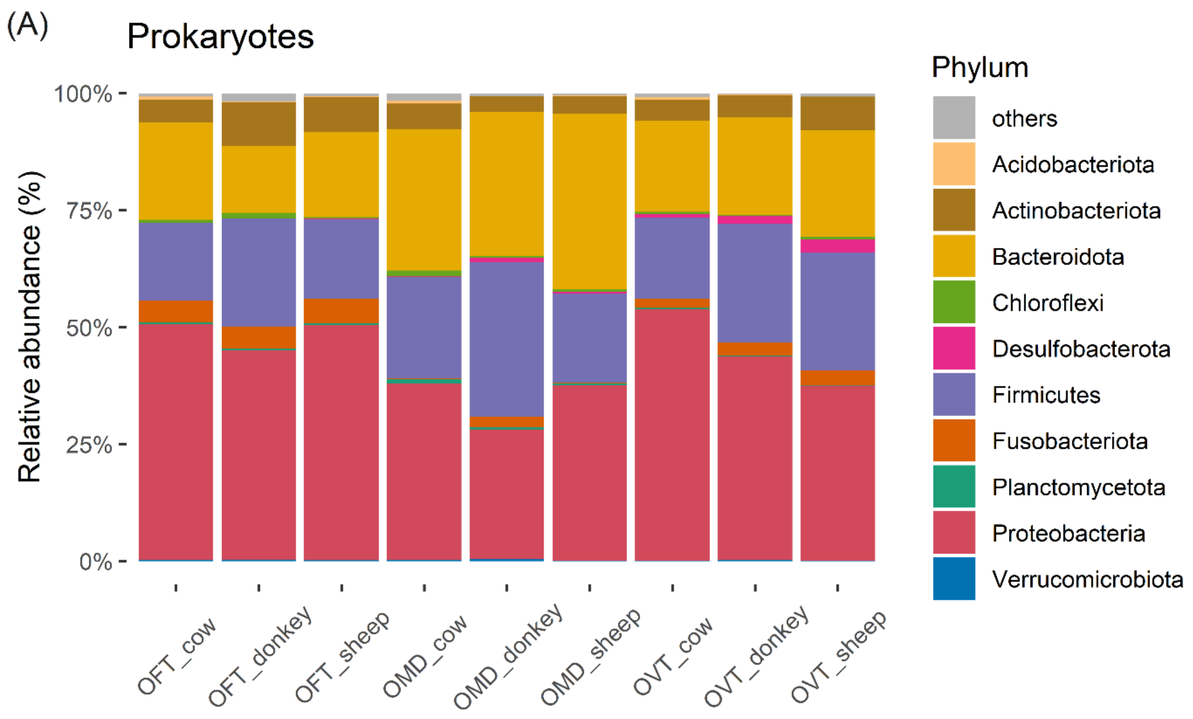

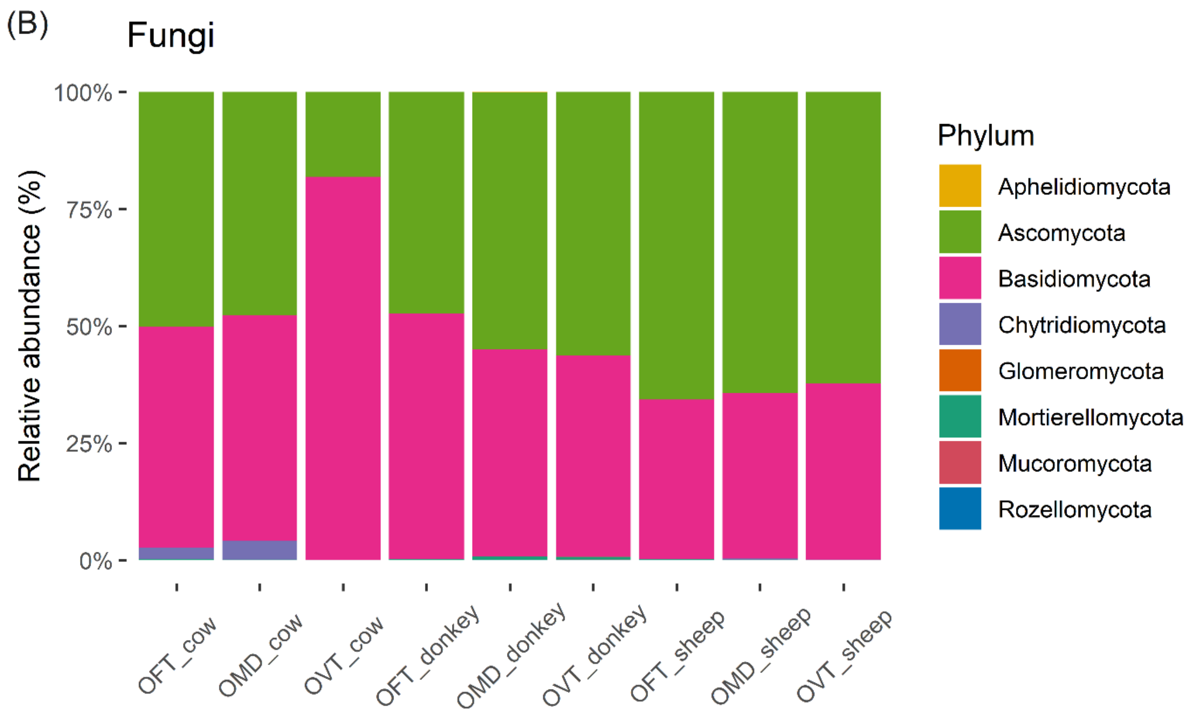

Figure S5. Stacked bar chart of the ten most abundant gut prokaryotes (A) and fungi (B) at the phylum level (OFT: *O. fracticornis*; OMD: *O. medius*; OVT: *O. verticicornis*).

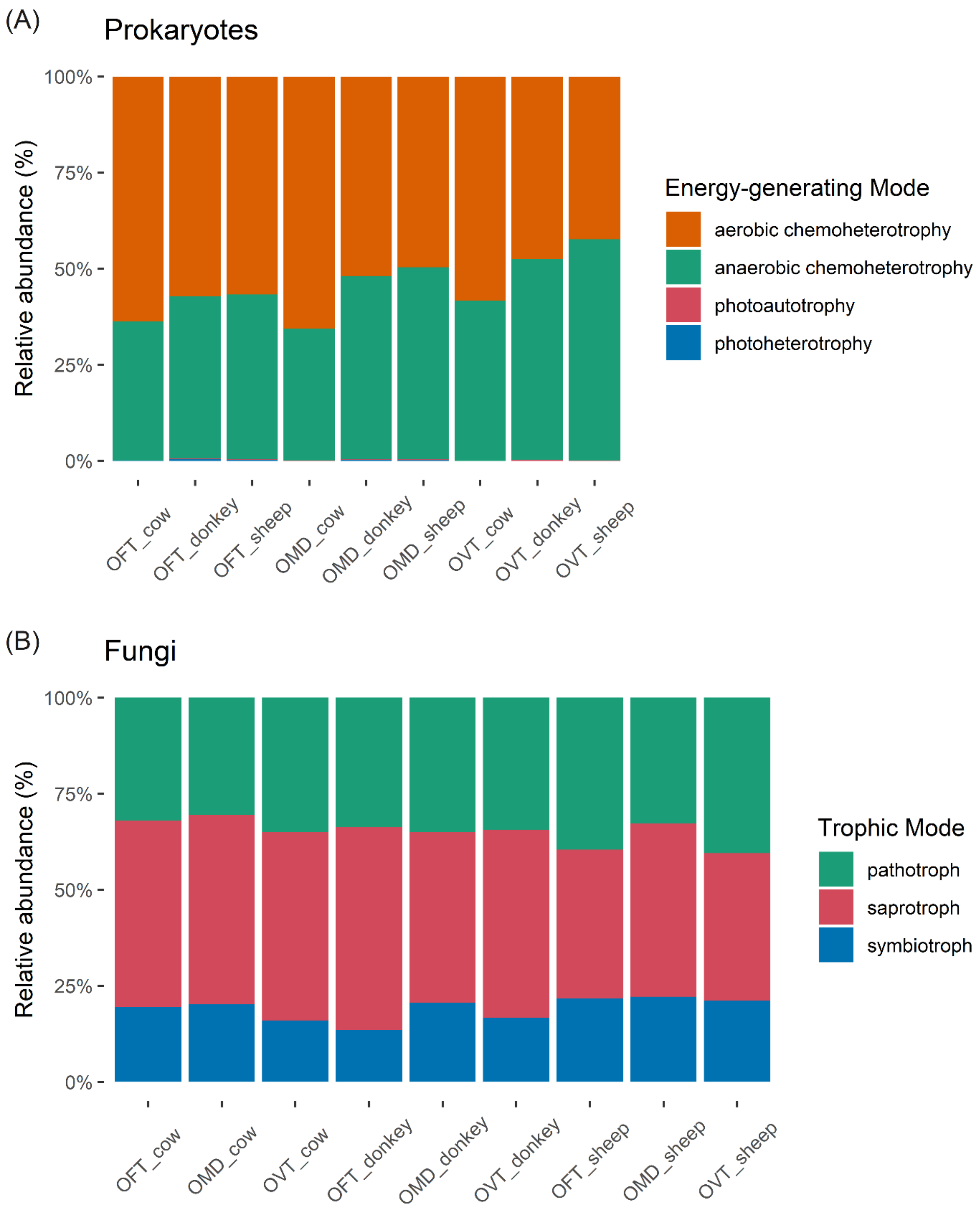

Figure S6. Stacked bar chart of the prokaryotic energy-generating modes (A) and fungal trophic modes (B) (OFT: *O. fracticornis*; OMD: *O. medius*; OVT: *O. verticicornis*).

Table S2. Prokaryotic alpha diversity values for all individuals (i.e. 90 dung beetles).

Table S3. Fungal alpha diversity values for all individuals (i.e. 90 dung beetles).
